# Safe dissection and complication avoidance for L1–2 interbody placement via a lateral access approach

**DOI:** 10.3171/2022.3.FOCVID2221

**Published:** 2022-07-01

**Authors:** David S. Xu, Gabriella M. Paisan, Joelle N. Hartke, Gennadiy A. Katsevman, Juan S. Uribe, Laura A. Snyder

**Affiliations:** 1 Department of Neurosurgery, Ohio State University Wexner Medical Center, Columbus, Ohio; and; 2 Department of Neurosurgery, St. Joseph’s Hospital and Medical Center, Barrow Neurological Institute, Phoenix, Arizona

**Keywords:** diaphragm dissection in spine surgery, lateral access approach, lateral transpsoas approach, thoracolumbar junction

## Abstract

The lateral access approach for L1–2 interbody placement or other levels at or near the thoracolumbar junction may be difficult without proper knowledge and visualization of anatomy. Specifically, understanding where the fibers of the diaphragm travel and avoiding injury to the diaphragm are paramount.

The video can be found here: https://stream.cadmore.media/r10.3171/2022.3.FOCVID2221

## Transcript

This video will demonstrate safe dissection and complication avoidance for L1–2 interbody placement via a lateral access approach.

### 0:30

A woman in her mid-70s presented with burning low-back pain, radiating to her left hip, occasionally extending into her anterior left thigh. She reported progressive bilateral leg weakness, worsening with ambulation. On physical examination, she had 5/5 strength in bilateral lower-extremity muscle groups. She had no abnormal reflexes.

### 0:54

Preoperative standing scoliosis radiographs demonstrated the patient’s earlier L2–S1 fusion and fixation, with a broken left S1 screw. Her spinal vertical alignment was 3 cm, lumbar lordosis was 57°, her pelvic incidence was 82°, and her pelvic tilt was 45°.

### 1:16

Preoperative MRI showed prominent marrow edema in the L1–2 vertebral bodies with moderate central canal stenosis. There was moderately severe left and moderate right neural foraminal narrowing.

### 1:28

Preoperative flexion and extension radiographs showed no instability at the L1–2 disc space but revealed a vacuum disc at this level.

### 1:36

After review of the patient’s history, physical examination findings, and imaging, as well as discussion with the patient, we decided to proceed with a right lateral L1–2 transpsoas interbody fusion with use of morselized allograft. Due to the painful nature of the patient’s multiple earlier posterior operations, including fixation and fusion, her history of screw breakage, as well as earlier cerebrospinal fluid leak, the patient desired to avoid posterior fusion and fixation. We discussed the risks associated with a stand-alone lateral interbody fusion, including subsidence and the need for further surgery, and the patient demonstrated understanding and desired to proceed.

### 2:20 Initial Incision.

The video shows the initial incision and the subcutaneous fat beneath.

### 2:24 Incision Extension and Fascial Incision.

The Bovie is used to extend the incision as well as incise the fascia. Underneath, further fat can be seen.

### 2:32 Hemostasis.

Hemostasis of the fat and fascia is important to prevent postoperative hematoma as well as blood running down into the surgical field once the retractor is in place. The Bovie can also be used as a dissection instrument to start pushing away the fat from the muscle fibers.

### 2:48 Abdominal Muscle Fiber Visualization.

Tonsillar forceps are used to spread the various layers of the lateral abdominal muscle. One can identify the layers of muscle due to the directionality of the fibers. The external oblique muscle will be encountered first. Its fibers run downward and medially. Deep to this, the internal oblique muscle will be seen, with its fibers traveling upward and medially to insert into the ribs and linea alba. The last muscular layer is the transversus abdominis, the fibers of which run horizontally.^1^

### 3:18 Abdominal Muscle Fiber Dissection.

The subcostal and iliohypogastric nerves, which originate from the T12 and L1 nerve roots, respectively, supply motor innervation to the muscles of the anterior abdominal wall. Copious dissection of the muscle or use of the Bovie in this plane may cause injury to these nerves, which may result in abdominal wall pseudohernia.^1^ With the use of a blunt finger dissection and poor visualization, injury beneath to the diaphragm and the pleura may occur, placing patients at risk of pneumothorax and pleural effusion.^2^

### 3:47 Entry Into Retroperitoneal Space, Identification of Diaphragm.

After the layers have been spread, the retroperitoneal space is entered, and the fibers of the diaphragm can be seen. They are larger, covered by a bright white sheen muscle fascia, and travel in a different direction than the fibers of the abdominal wall.

### 4:05 Sweeping the Fibers of the Diaphragm.

If the most inferior fibers of the diaphragm cannot be well visualized, one can use a finger to feel the fibers in the field and track them by feeling inferiorly to where the bottom edge of the diaphragm curves to its attachment sites. Then, these fibers can be swept superiorly toward the head, to give the surgeon access to the psoas muscle without diaphragmatic injury.^3^

### 4:33 Direct Psoas Muscle Visualization.

Now long retractors can be used to retract the diaphragm and the other contents of the retroperitoneal space anteriorly, as well as provide direct visualization of the psoas muscle underneath. At L1–2, the psoas forms a thin muscular layer with contributors to the lumbar plexus, in particular the ilioinguinal and genitofemoral nerves, which may be injured when spreading the muscle.^4^ Direct visualization of the psoas muscle and avoidance of these branches, if identified, provides for a safe retractor docking without injury to the nerves or other retroperitoneal contents.^5^

### 5:06 Evaluating the Diaphragm.

One can visualize the expansion of the diaphragm and the lungs below as the diaphragm tracks in and out of the field with each breath.

### 5:13 Retractor Docking.

The retractor can be safely docked after dilation through the psoas muscle with tubular dilators and careful neurophysiological (or electromyographic) monitoring of these dilators, minimizing the risk of intraoperative injury. A shim in the posterior portion of the disc space prevents migration of the retractor.

### 5:31 Cutting the Disc Annulus.

The disc annulus is cut using a retractable blade to avoid injury when coming in and out of the retractor. Care is taken not to cut too close to the posterior shim because this may dislodge the retractor. Cutting the annulus too anteriorly is avoided to prevent inadvertently cutting the anterior longitudinal ligament. The anterior longitudinal ligament is important for limiting extension and axial rotation, and inadvertent rupture of the ligament leads to segmental hypermobility, which may lead to facet degeneration at this and adjacent levels.^6^

### 6:00 Disc Removal.

Then the disc can be removed with a combination of pituitary rongeurs, Kerrison rongeurs, and curettes.

### 6:05 Trial Placement.

Trials can be used to start determining the goal interbody size.

### 6:08 Curettage.

The curette is used to scrape the cartilaginous endplates to prepare the bone for fusion.

### 6:14 Interbody Placed.

A titanium interbody graft packed with morselized allograft is placed under anteroposterior and lateral fluoroscopic guidance.

### 6:22 Anterior Retractor Removal.

The anterior retractor is removed for improved visualization of the graft. Pieces of allograft that extruded from the interbody are removed from the field.

### 6:31 Further Hemostasis and Irrigation.

Floseal is placed in the wound to promote hemostasis and then partially irrigated out with antibiotic-impregnated saline to prevent infection.

### 6:40 Vancomycin Placement.

Vancomycin powder is also placed in the wound to prevent infection.

### 6:44 Retractor Removal.

Slow retractor removal provides visualization of the psoas muscle fibers falling back into position with minimal injury, the expansion of the diaphragm without injury, and no evidence of blood pooling in the surgical field.

### 6:58

Postoperative MRI findings indicated that the patient no longer had canal or foraminal stenosis at L1–2.

### 7:06

Postoperative scoliosis radiographs demonstrated the interval placement of the L1–2 interbody. The patient’s pelvic parameters had slightly improved. Her spinal vertical axis was 2 cm positive, lumbar lordosis was 64°, pelvic incidence was 82°, and pelvic tilt was 43°. Most importantly, the patient’s left anterior thigh pain improved within the 1st week of surgery, and her preoperative back pain had improved by the time of her 6-week postoperative visit.

## Acknowledgments

We thank the staff of Neuroscience Publications at Barrow Neurological Institute for assistance with manuscript and video preparation.

## Disclosures

Dr. Snyder is a consultant for Medtronic, Globus, and NuVasive and obtains research funding from Biogen. Dr. Xu is a consultant for NuVasive. Dr. Uribe receives consulting fees and royalties from NuVasive Medical, Inc., and is a consultant for Masonix, Inc., and SI Bone, Inc.

## Author Contributions

Primary surgeon: Snyder. Assistant surgeon: Xu. Editing and drafting the video and abstract: Snyder, Xu, Paisan, Hartke, Uribe. Critically revising the work: all authors. Reviewed submitted version of the work: all authors. Approved the final version of the work on behalf of all authors: Snyder. Supervision: Snyder, Uribe.
